# Bacterial and fungal communities regulated directly and indirectly by tobacco-rape rotation promote tobacco production

**DOI:** 10.3389/fmicb.2024.1418090

**Published:** 2024-06-14

**Authors:** Lu Liu, Qi Miao, Yingxin Guo, Chen Wang, Junwei Sun, Zhiyong Fan, Dexun Wang, Yanxia Hu, Junying Li, Zhenling Cui

**Affiliations:** ^1^State Key Laboratory of Nutrient Use and Management, College of Resources and Environmental Sciences, National Academy of Agriculture Green Development, China Agricultural University, Beijing, China; ^2^Key Laboratory of Nutrient Cycling and Arable Land Conservation of An Hui Province, Soil and Fertilizer Research Institute, Anhui Academy of Agricultural Sciences, Hefei, China; ^3^Yunan Dali Tobacco Company, Dali, China; ^4^Yunnan Academy of Tobacco Agriculture Science, Kunming, China

**Keywords:** tobacco-rape rotation, tobacco yield, soil chemical properties, bacterial community, fungal community

## Abstract

Tobacco continuous cropping is prevalent in intensive tobacco agriculture but often leads to microbial community imbalance, soil nutrient deficiency, and decreased crop productivity. While the tobacco-rape rotation has demonstrated significant benefits in increasing tobacco yield. Microorganisms play a crucial role in soil nutrient cycling and crop productivity. However, the internal mechanism of tobacco-rape rotation affecting tobacco yield through microbe-soil interaction is still unclear. In this study, two treatments, tobacco continuous cropping (TC) and tobacco-rape rotation (TR) were used to investigate how planting systems affect soil microbial diversity and community structure, and whether these changes subsequently affect crop yields. The results showed that compared with TC, TR significantly increased the Shannon index, Chao1 index, ACE index of bacteria and fungi, indicating increased microbial α-diversity. On the one hand, TR may directly affect the bacterial and fungal community structure due to the specificity of root morphology and root exudates in rape. Compared with TC, TR significantly increased the proportion of beneficial bacterial and fungal taxa while significantly reduced soil-borne pathogens. Additionally, TR enhanced the scale and complexity of microbial co-occurrence networks, promoting potential synergies between bacterial OTUs. On the other hand, TR indirectly changed microbial community composition by improving soil chemical properties and changing microbial life history strategies. Compared with TC, TR significantly increased the relative abundance of copiotrophs while reduced oligotrophs. Notably, TR significantly increased tobacco yield by 39.6% compared with TC. The relationships among yield, microbial community and soil chemical properties indicated that planting systems had the greatest total effect on tobacco yield, and the microbial community, particularly bacteria, had the greatest direct effect on tobacco yield. Our findings highlighted the potential of tobacco-rape rotation to increase yield by both directly and indirectly optimizing microbial community structure.

## 1 Introduction

Tobacco (*Nicotiana tobacum* L.) is a significant economic crop grown in more than 100 countries around the world, with China accounting for about a third of the world’s tobacco production (Yang et al., 2015; Reichert et al., 2019). Growing economic demands and limited arable lands have propelled tobacco continuous cropping (TC) as a prevailing practice in China’s intensive and large-scale agriculture. However, the prolonged cultivation of a single species over many years often results in adverse consequences, including the deterioration of soil properties and imbalance of microbial community, ultimately leading to a decrease in crop yields. After 5 years of continuous tobacco cultivation, there is a substantial decrease of 35% in yield and 47.6% in output value compared to the initial crop (Jin et al., 2024). A critical facet of effective agricultural management, particularly in mitigating the challenges posed by continuous cropping, is the implementation of crop rotation. Crop rotation leads to an average increase in crop yields by 20% when contrasted with continuous cropping (Linh et al., 2015; Zhao et al., 2020). In particular, tobacco-rape rotation (TR) has a significant positive impact on tobacco yield due to the special root exudates and efficient nutrient transformation of rape (Fang et al., 2016; Yao et al., 2021). Therefore, it is of great significance to explore how TR alleviates the obstacles of TC by reducing the deterioration of soil properties and the imbalance of microbial community so as to improve tobacco yield.

Soil microbes participate in some key processes of the agroecosystem cycle and play an important role in driving soil organic carbon (SOC) decomposition, inhibiting soil-borne pathogens, maintaining the soil nutrient cycle, and enhancing crop productivity (Guo et al., 2021). Planting systems can induce substantial changes in bacterial and fungal communities. TC leads to an intensified and constant exposure of microbial communities to root exudates from the tobacco, potentially resulting in an imbalance in the microbiota (Fu et al., 2017; Liu et al., 2020). The abundance of beneficial bacteria, such as *Arthrobacter* and *Lysobacter*, decrease and soil-borne pathogens increase (Wu et al., 2017; Wang C. et al., 2022). The effect of TC on microbial co-occurrence networks showed that the soil from TC has a less complex network (fewer modules, nodes, and connectivity) and more competition or antagonism existed among bacterial species (Chen et al., 2018). While crop rotation emerges as a potent modulator of soil microbial diversity and the ecological relationships among community members. The plant species turnover across space and time in crop rotation systems can increase microbial diversity and balance soil microbial communities (Zavaleta et al., 2010; Wang P. et al., 2022). Compared to rice, rape has a larger and deeper root system, and special exudates of the rape root system can resist tobacco black shank (Yang et al., 2012). However, there is little research on how TR affects the microbial community structure through microbial α diversity, community composition, and microbial occurrence network.

Changes in soil chemistry caused by the shift of planting systems affect microbial community composition. For example, SOC, available nitrogen (AN), and available potassium (AK) are the dominant factors of bacterial and fungal communities in potato-green manure rotation system, while pH is highly correlated with bacterial and fungal community structure in potato continuous cropping system (Wang X. et al., 2022). Moreover, soil microbial community composition is usually regulated by the availability of resources such as C, N and phosphorus (P), which is mainly reflected in the change of microbial life history strategies (Sinsabaugh et al., 2009). The classification of microorganisms based on their life-history characteristics simplifies the complexity of microbial community composition (Chen et al., 2021). A classic life-history classification of microbes is the copiotroph-oligotroph (*r*/*K* strategist) dichotomy (Fierer et al., 2012). Copiotrophs, characterized as r-strategists, thrive in resource-rich conditions, emphasizing rapid growth. In contrast, oligotrophs, or *K*-strategists, prioritize resource efficiency at the expense of growth rate (Koch, 2001). It was found that copiotrophs (*Bacteroidetes*, *Gemmatimonadetes*, *Proteobacteria*, *Mortierellomycota*) thrive in soils with high SOC and inorganic nutrient availability (Shao et al., 2021). In addition, the beneficial and harmful taxa in the soil are also regulated by nutrient availability. Soil beneficial floras were positively correlated, and pathogenic fungal taxa were negatively correlated with the SOC and mineral nutrients (Wang X. et al., 2022). Continuous cropping usually has adverse effects on soil nutrient dynamics. On the one hand, continuous cropping leads to the selective absorption of soil nutrients by crops, resulting in the discernible imbalance and deficiency of crucial nutrients (Aller et al., 2017; Agomoh et al., 2021). On the other hand, continuous cropping system significantly diminishes nutrient chelation capacity and exacerbates nutrient loss (Jiang et al., 2022). While considerable evidences support the positive impact of crop rotation on soil properties. For instance, crop rotation has positive implications for SOC formation and potential N mineralization (Ai et al., 2015; Liu et al., 2022). Especially compared with TC, tobacco-wheat rotation and tobacco-potato rotation, TR has outstanding advantages in improving soil nutrient conversion, increasing SOC, TN, AN, AP, and AK (Wang et al., 2015). However, how TR regulates bacterial and fungal community composition by affecting soil nutrient availability has been unclear.

The effects of soil physicochemical properties and microbial community on crop yield have been studied extensively (Kelley et al., 2003; Iqbal et al., 2022). Rational soil structure and SOC content can improve soil pH and nutrient cycling in the rhizosphere, and ultimately promote tobacco growth (Wright and Hons, 2005). Crop rotation generally increases pH (Wang P. et al., 2022), but in the long run, the potential effect of organic acids secreted by rape roots on soil pH and nutrient cycling in TR systems remains uncertain. In addition, microorganisms have been shown to be the drivers of productivity in farmland ecosystems. Soil microbiomes can influence crop productivity in many ways, such as directly affecting root-associated organisms or indirectly changing the nutrient cycling rates and the partitioning of resources (Van Der Heijden et al., 2008). However, the underlying mechanism of how TR improves tobacco yield by directly or indirectly regulating microbial communities is still unclear. Therefore, we will study from the following aspects: (1) the mechanism of TR affecting microbial community (bacteria and fungi) from the perspective of soil chemistry, microbial diversity, community composition, co-occurrence networks; (2) how soil chemical nutrient availability and microbial communities driven by planting systems influence tobacco yield and their contribution to tobacco yield. Understanding the internal mechanism of tobacco-rape rotation driving yield improvement is crucial not only for advancing our knowledge of sustainable agricultural practices but also for offering practical insights that can optimize tobacco cultivation strategies, particularly in regions where economic and land resource constraints dictate farming practices.

## 2 Materials and methods

### 2.1 Site description

A long-term field experiment was established in 2007 in Yuxi City, Yunnan Province, China (N 24°18′, E 102°38′). The climate for this region was characterized as subtropical monsoon climate, with an average annual precipitation of 773 mm, with an average annual temperature of 15.6°C. The soil in the test field was typical of the sandy red soil found in Yunnan province as the main type of tobacco planting soil. Based on the USDA soil taxonomy, the red soil was classified as an Ultisol (USDA, 2014). The soil was composed of 28% of sand.

### 2.2 Field experiment design

Two farmland management systems were established: tobacco continuous cropping (TC) and tobacco-rape rotation (TR). The field trial design was a randomized complete block design with two treatments and three replications, for a total of six plots. The size of each plot was15 m × 10 m (length × breadth). Breeding seedlings to obtain healthy tobacco seedlings in March and transplanting seedlings occurred in April. The planting density of tobacco was 16,500 plants/ha, and plant spacing was 50 cm × 120 cm. During the growing period from planting in March to harvesting in September each year, tobacco in continuous cropping and tobacco-rape rotation systems received the same amount of compound fertilizer (N: 124 kg/ha, P_2_O_5_: 124 kg/ha, K_2_O: 309 kg/ha). One-third of compound fertilizer was applied as base fertilizers before transplanting, and the remaining two-thirds was applied 30 days after transplanting. The tobacco cultivar K326 for the test was provided by Zhongyan Tobacco Seed Co., Ltd., China. Tobacco residues were removed after the harvest. Under the TR system, after the tobacco harvest in September, six holes were set per square meter, and three plants of rape (A35 variety) were planted in each hole. And 75 kg/ha compound fertilizer (12:6:24) dissolved in water and applied by sprinkler irrigation. Rape residues were removed after the rape harvest in March. The TC system was fallow during rapeseed cultivation.

### 2.3 Sample collection

At the time of tobacco harvesting, three representative tobacco plants were randomly taken from each plot and the specific method of harvesting in accordance with Tang et al. (2020). The tobacco leaves were baked to calculate tobacco yield. Soil samples were collected using a soil corer (inner diameter of 5 cm) in September 2020 after tobacco harvesting. Thirty soil cores were collected per plot with a probe to a depth of 20 cm. Every 10 cores were mixed into one composite sample. Each plot included three composite samples, equivalent to 9 replicates per treatment. The samples were then stored in airtight polypropylene bags and were rapidly transported to the laboratory where each soil sample was sieved with a 2 mm mesh and divided into two portions. One part was stored at −80°C for DNA extraction, and the other was used for the for the analysis of soil properties.

### 2.4 Soil properties analysis

The ring knife method was used to determine the soil bulk density and soil porosity. The DIK1150 three-phase instrument (Beijing Haifuda Technology Co., LTD.) was used to determine the soil three-phase ratio which equals soil solid volume: soil liquid volume: soil gas volume. Soil pH was measured in a soil–water suspension (1:2.5) using a pH meter. Soil electrical conductivity (EC), the indicator of soil soluble salt, was measured in a soil–water suspension (1:5) using an electric conductometer. The carbonate in the soil was removed by hydrochloric acid. SOC and total nitrogen (TN) were measured using an elemental analyzer (Vario EL III, Elementar, Germany). Soil available nitrogen (AN) levels, including ammonium, nitrate, and easily decomposable and hydrolysable organic nitrogen, were determined using alkali distillation (Page et al., 1982). Total P (TP) and available P (AP) were extracted with HF-HNO_3_-HClO_4_ and sodium bicarbonate, respectively, and then determined by the molybdenum-blue method (Page et al., 1982). Total K (TK) and available K (AK) were extracted with HF-HNO_3_-HClO_4_ and ammonium acetate, respectively, and then determined by flame photometry (Halajnia et al., 2009).

### 2.5 DNA extraction and illumina sequencing

DNA was extracted from fresh soil samples (0.5 g per sample) using MP FastDNASPIN Kits (MP Biomedicals, Solon, OH, United States). DNA quality and integrity were examined using 1% agarose gel electrophoresis. Bacterial 16S rRNA gene fragments were performed using the general bacterial primers 338F–806R, which are specific to the V3–V4 hypervariable region. The ITS region was targeted with the primers ITS1F–ITS2. Sequencing libraries were generated using TruSeq Nano DNA LT Library Prep Kit (Illumina, United States) following the manufacturer’s recommendations. High-throughput sequencing of 16S rRNA and fungal ITS genes were carried out using an Illumina HiSeq2500 platform, and 250 bp paired-end reads were generated.

The raw reads were denoised, dereplicated, and clustered using the Quantitative Insights into Microbial Ecology (QIIME) pipeline (Caporaso et al., 2010). The resultant high-quality sequences were clustered into operational taxonomic units (OTUs) at 97% similarity using the UPARSE algorithm (Edgar, 2013), and the most abundant sequence from each OTU was selected as its representative sequence. Taxonomic classification of representative sequences from individual OTUs was performed by the RDP classifier (Wang et al., 2007). In order to compare relative differences between samples, a randomly selected subset of 88,336 and 32,620 sequences per sample for prokaryotes and fungi, respectively, was subjected to downstream analyses.

### 2.6 Statistical analysis

The Shannon index, Chao1 index, and ACE index were used to evaluate the α-diversity of bacterial and fungal communities (Zhou et al., 2021). *T*-tests were conducted using SPSS 20.0 for Windows software (SPSS Inc., Chicago, IL, United States) to discern the differences between TC and TR in α-diversity, the relative abundance of bacteria or fungi at different taxonomic levels, soil physicochemical properties, and tobacco yield. The analysis of non-metric multidimensional scaling (NMDS) based on the Bray–Curtis similarity matrix was used to explore the differences between TR and TC in soil microbial community composition in R (Version 3.4.1). For microbial networks, only OTUs with relative abundance larger than 0.01%. The relationships between OTUs with Spearman’s correlation coefficient *r* > 0.7 or *r* < −0.7 and *p* < 0.01 were selected for network construction. Then the R “igraph” package was used for the construction of co-occurrence network and calculation of network topological properties. The parameters describing network topological properties used in this study included nodes, edges, average degree, network diameter, average path length, clustering coefficient, modularity, density, and positive and negative links (Ma et al., 2016). Visualization of the co-occurrence network was performed by using Gephi. Additionally, redundancy analysis (RDA) was conducted using the vegan package in R (Version 3.4.1) to unveil the relationships between soil microbial communities and soil chemical properties. The relationships between soil microbial community composition at the phylum level and tobacco yield or soil properties were determined with Pearson correlations using OriginPro 2024 (Origin Lab Corp., Northampton, MA, United States). To unravel the complex interplay among soil chemical properties, microbial communities, and tobacco yield, structural equation modeling (SEM) was performed using AMOS 23.0. Microbial α-diversity, NMDS and *r*-strategy taxa (or copiotrophs) were hidden variables of microbial communities including bacteria and fungi. The data were meticulously fitted to the models using the maximum likelihood estimation method. The SEM was rigorously assessed for fit using the χ^2^-test, comparative fit index (CFI), and the root square mean error of approximation (RSMEA). These metrics collectively provided a robust evaluation of the model.

## 3 Results

### 3.1 Soil properties and tobacco yield

Compared with TC, TR had no significant effect on soil physical properties including soil three-phase ratio, soil porosity and soil bulk density (*p* > 0.05, Supplementary Table S1), but influenced significantly on soil chemical properties (Table 1). Compared with TC, TR significantly decreased soil pH by 4.7%, while significantly increased SOC, TN, TK, AN, and AK by 20.2, 24.7, 9.6, 32.3, and 5.7%, respectively (*p* < 0.05, Table 1). In addition, TR also affected the tobacco yield. Compared with TC, TR increased the tobacco yield by 39.6% (*p* < 0.05, Table 1). There was no significant difference in tobacco quality between TC and TR (Supplementary Table S2).

**Table 1:** Soil chemical properties and tobacco yield under tobacco continuous cropping and tobacco-rape rotation^a^.

Planting systems	pH	EC	SOC	TN	TP	TK	AN	AP	AK	Yield
		(μs cm^−1^)	(g kg^−1^)	(g kg^−1^)	(g kg^−1^)	(g kg^−1^)	(mg kg^−1^)	(mg kg^−1^)	(mg kg^−1^)	(kg ha^−1^)
TC	6.77 ± 0.14 A	96.9 ± 15.8 A	5.25 ± 0.22 B	0.68 ± 0.05 B	0.52 ± 0.03 A	3.61 ± 0.57 B	51.1 ± 2.63 B	97.8 ± 15.7 A	148 ± 18.8 B	1905 ± 450 B
TR	6.4 ± 0.11 B	89.8 ± 9.64 B	6.31 ± 0.74 A	0.85 ± 0.03 A	0.54 ± 0.03 A	3.96 ± 0.56 A	67.6 ± 5.2 A	92.6 ± 7.57 A	156 ± 9.34 A	2,655 ± 450 A

a Values are means ± standard errors (*n* = 9). EC, electric conductivity; SOC, soil organic carbon; TN, total nitrogen; TP, total phosphorus; TK, total potassium; AN, alkaline hydrolysis nitrogen; AP, available phosphorus; AK, available potassium. Different uppercase letters stand for statistical differences (*t* test, *p* < 0.05) between tobacco continuous cropping (TC) and tobacco-rape rotation (TR).

### 3.2 Microbial community diversity and community composition

**Figure 1:**
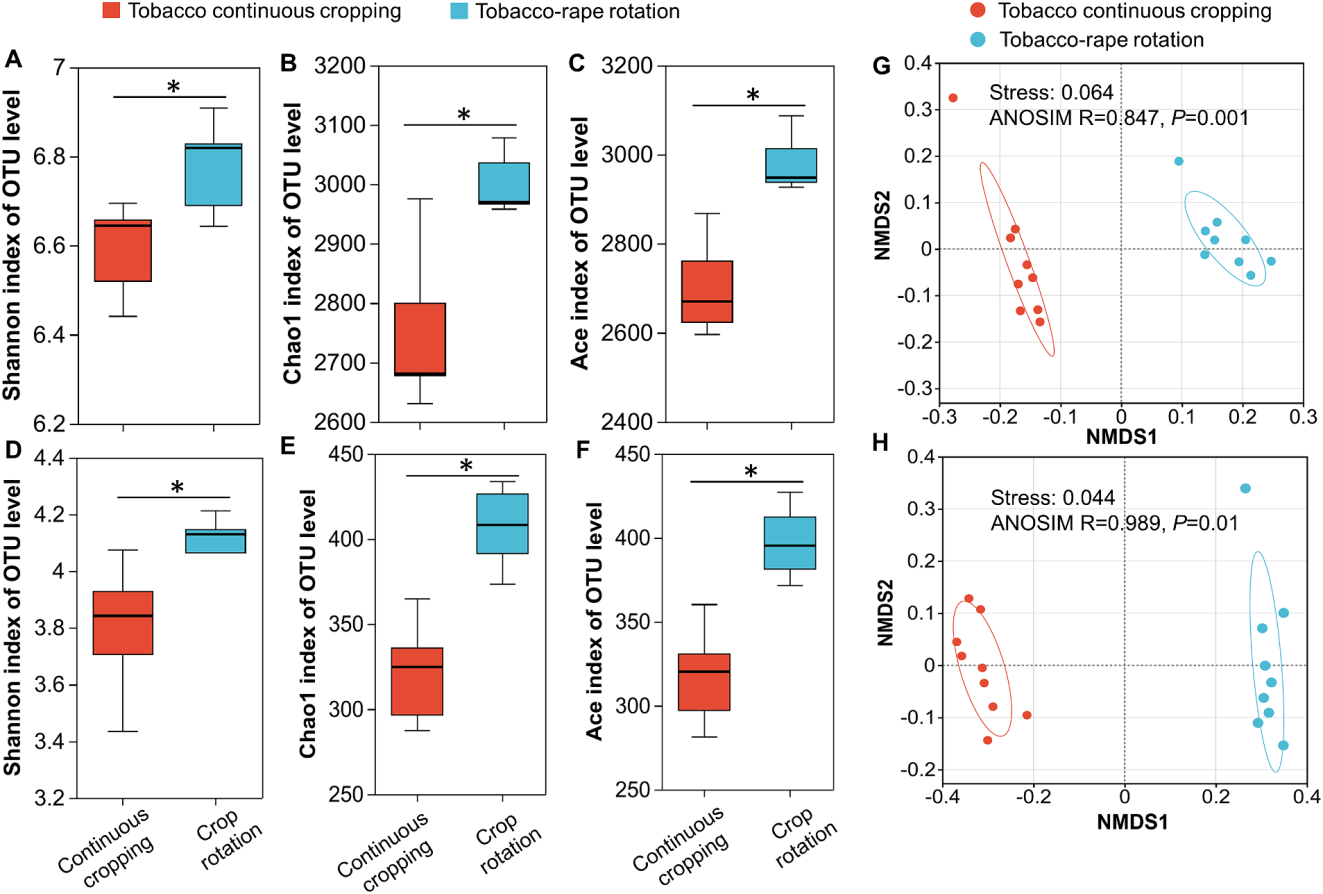
The alpha-diversity indexes and non-metric multidimensional scaling (NMDS) of bacteria **(A,B,C,G)** and fungi **(D,E,F,H)** under tobacco continuous cropping and tobacco-rape rotation. Asterisks (*p < 0.05) represent differences between tobacco continuous cropping and tobacco-rape rotation.

Shannon index, Chao1 index and ACE index were used to indicate α-diversity of bacterial and fungal communities. Compared with TC, TR increased significantly the Shannon index, Chao1 index, and ACE index of bacterial and fungal communities (*p* < 0.05, Figures 1A–F). To determine the effect of planting systems on soil microbial community structure, the overall structural changes of bacterial and fungal microbiota were analyzed using NMDS based on Bray-Curtis dissimilarities. NMDS ordinations revealed that there were significant differences in bacterial and fungal community structure between TC and TR (*p* < 0.05, Figures 1G,H).

**Figure 2:**
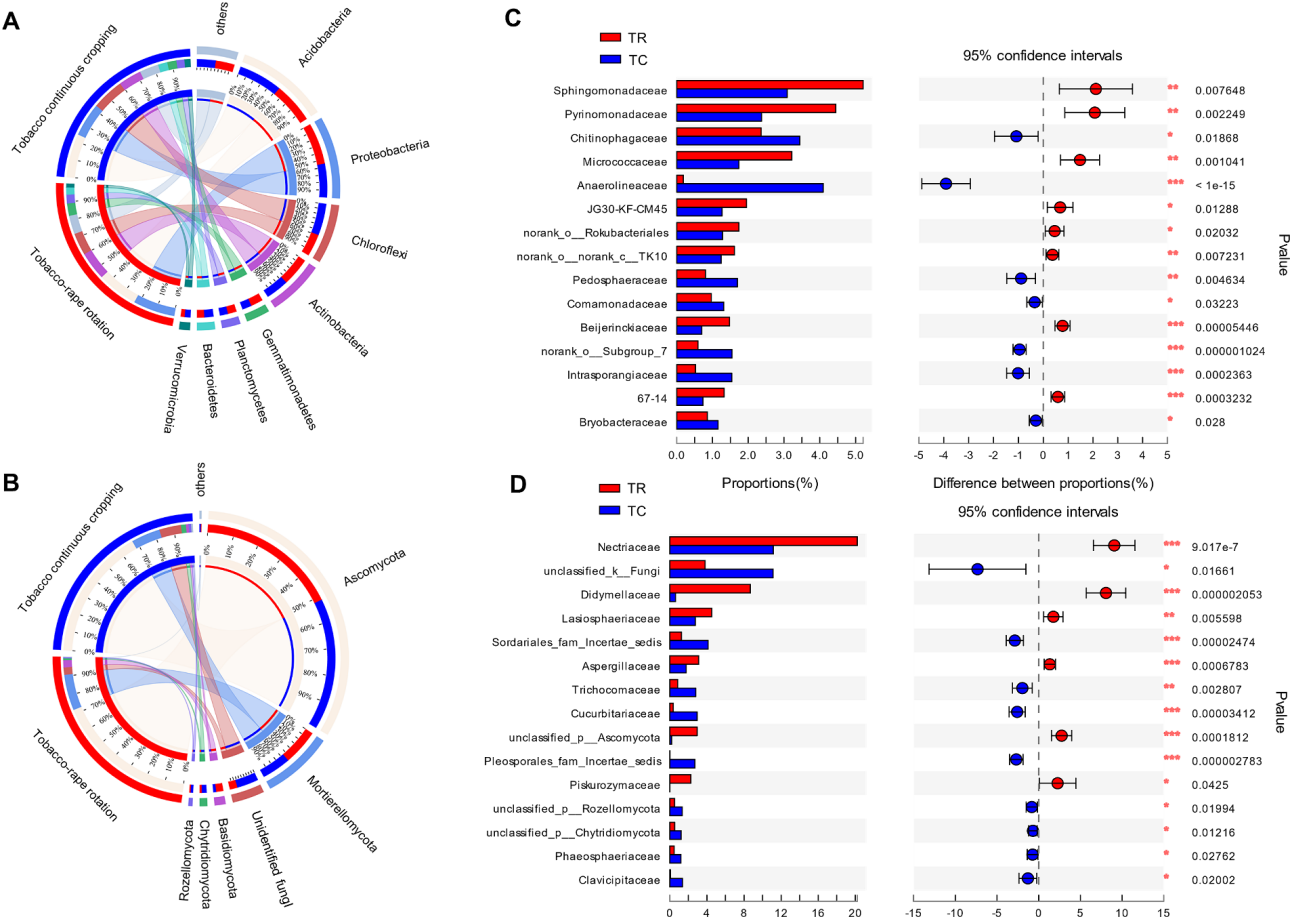
The relative abundance of bacterial **(A)** and fungal **(B)** at phylum, and the relative abundance of bacterial **(C)** and fungal **(D)** at family under tobacco continuous cropping (TC) and tobacco-rape rotation (TR).

*Acidobacteria*, *Proteobacteria*, *Chloroflexi*, and *Actinobacteria* were the dominant phyla of bacteria in both TC and TR systems (71–74% of the relative abundance of all OTUs) (Figure 2A). Compared with TC, TR increased significantly the relative abundance of *Proteobacteria*, *Actinobacteria* and *Gemmatimonadetes* by 19, 19.4 and 25.6%, respectively (*p* < 0.05, Figure 2A). While TR decreased significantly *Chloroflexi* and *Verrucomicrobia* by 43.9, 54.5%, respectively, compared to TC (*p* < 0.05, Figure 2A). Ascomycota (mean 70% of all OTUs) and *Mortierellomycota* (17%) were the dominant fungal phyla both treatments. Compared with TC, TR increased the relative abundance of *Ascomycota* and *Basidiomycota*, but decreased the relative abundance of *Rozellomycota* (*p* < 0.05, Figure 2B). The composition of bacteria and fungi at the family level showed that the TR increased significantly the beneficial bacterial taxa including *Sphingomonadaceae*, *Pyrinomonadaceae* and *Micrococcaceae* in community (*p* < 0.05, Figure 2C). And TR increased the beneficial fungal taxa including *Nectriaceae*, *Didymellace* and *Lasiosphaeriaceae* (*p* < 0.05, Figure 2D). Compared with TC, at the genus level of bacteria, TR increased significantly the relative abundance of *Sphingomonas* and *Arthrobacter* (*p* < 0.05, Supplementary Figure S1A). The relative abundance of *Mortierella* increased and *Fusarium* decreased at the genus level of fungi under TR system (*p* < 0.05, Supplementary Figure S1B).

### 3.3 Bacterial and fungal co-occurrence networks

**Figure 3:**
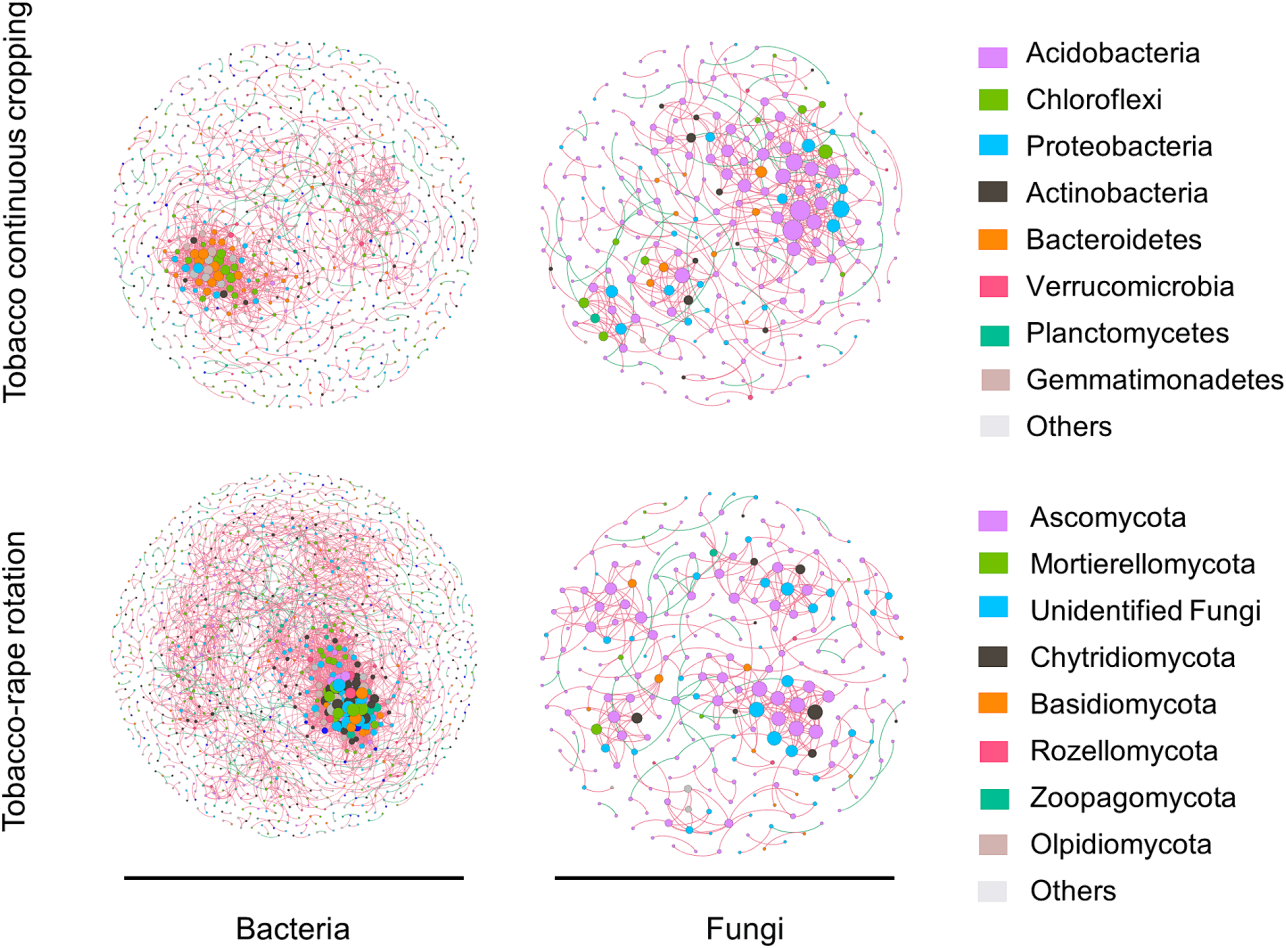
Effects of tobacco continuous cropping and tobacco-rape rotation on bacterial and fungal co-occurrence networks. The nodes of each network are colored according to phylum affiliation base on OTU and sized according to degree of connection. The edges connecting the nodes are represented by red lines to indicate co-occurrence interactions or green to indicate mutualistic exclusions.

Co-occurrence networks were constructed at the OTU level to identify the different co-occurrence patterns of the soil bacterial and fungal communities under TC and TR (Figure 3). The co-occurrence network topology properties of bacteria and fungi showed that compared with TC, TR increased the nodes, edges, average path distance, and network diameter of bacterial and fungal communities (Supplementary Table S3).

### 3.4 Correlation between soil chemical properties, microbial community and tobacco yield

**Figure 4:**
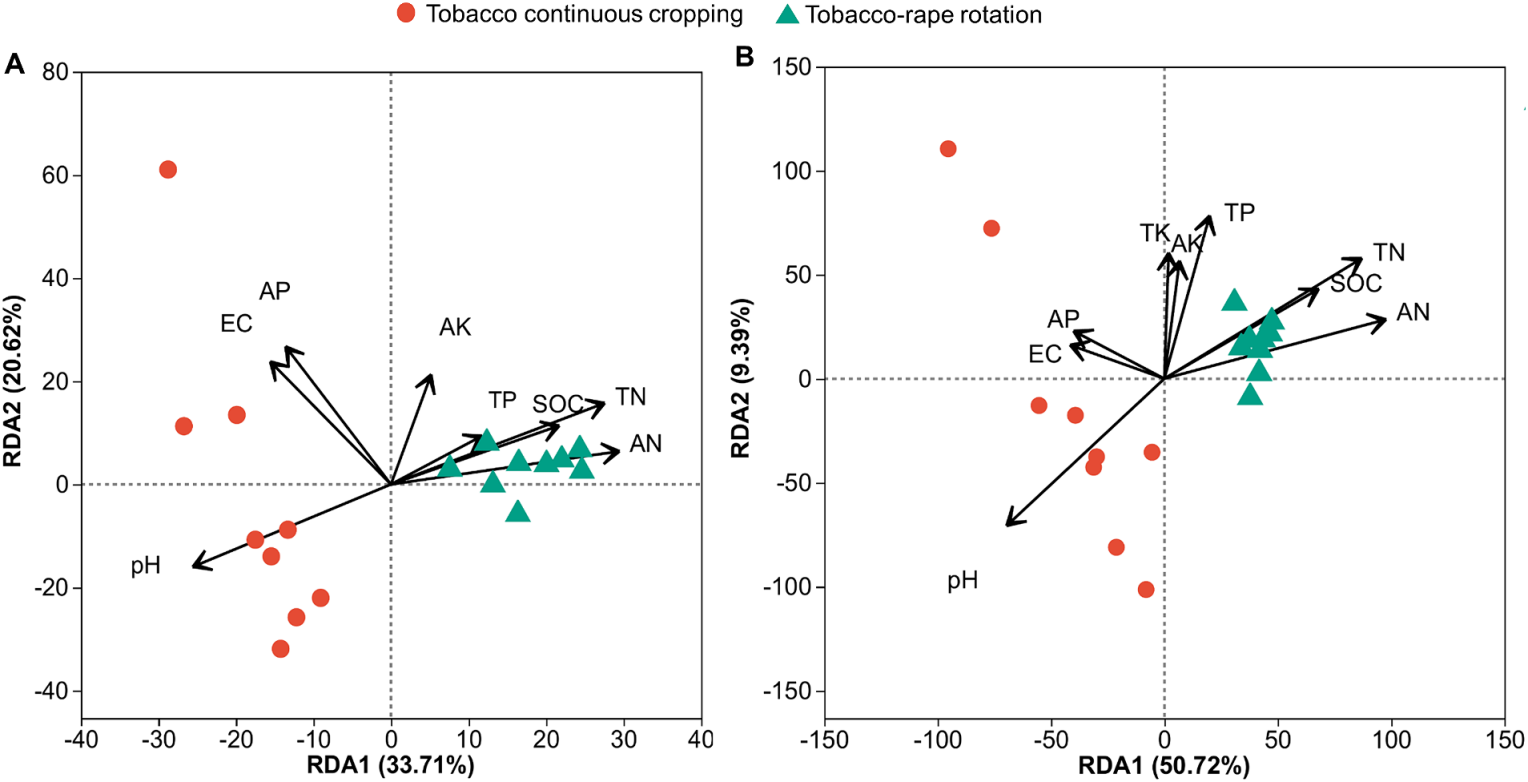
Redundancy analysis (RDA) demonstrating the relationships between soil properties and bacterial **(A)** or fungal **(B)** community structures under tobacco continuous cropping and tobacco-rape rotation. EC, electric conductivity; SOC, soil organic carbon; TN, total nitrogen; TP, total phosphorus; TK, total potassium; AN, alkaline hydrolysis nitrogen; AP, available phosphorus; AK, available potassium.

We used redundancy analysis (RDA) to assess the effects of soil chemical properties on the compositions of the bacterial and fungal communities. For the bacterial community, the first two axes together explained 54.3% of the total variation in the bacterial community (Figure 4A). Soil pH, EC, SOC, TN, AN, AP, and AK had significant effects on bacterial community structure. AK contributed the most to the change in bacterial community structure (Supplementary Table S4). For the fungal community, the first two axes together explained 60.1% of the total variation in the fungal community (Figure 4B). Soil pH, SOC, TN, AN, and AK had significant effects on the fungal community structure. TN contributed the most to the change in fungal community structure (Supplementary Table S4; Figure 4B).

**Figure 5:**
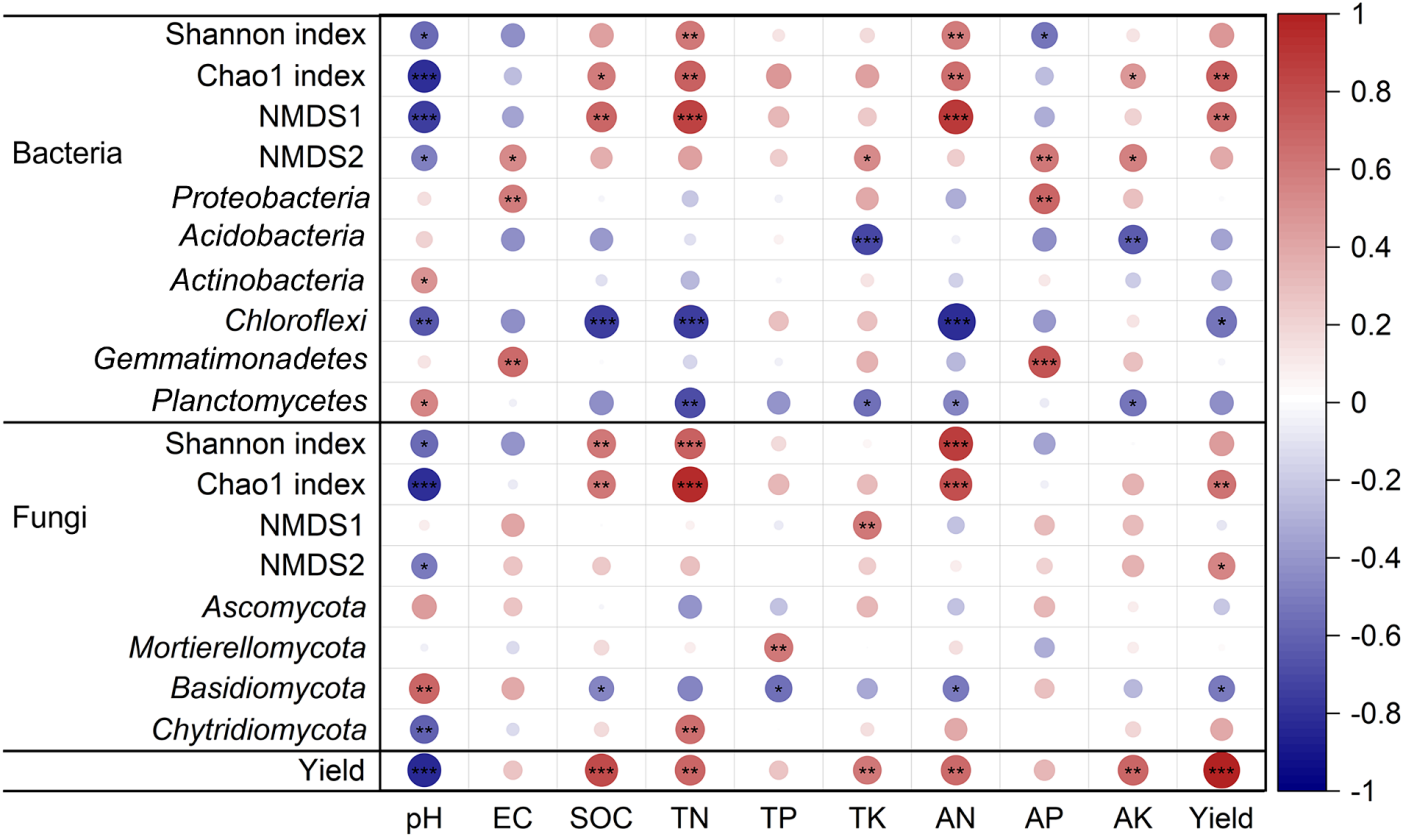
Pearson correlation coefficients between tobacco yield, soil properties, and soil microbial communities. *p < 0.05; **p < 0.01; ***p < 0.001. Red to blue color indicates positive to negative correlations.

In addition, the soil chemical properties had significant influence on microbial diversity (α and β diversity) and community composition at the phylum level (*p* < 0.05, Figure 5). SOC, TN, and AN were positively correlated with the Chao 1 index and NMDS1 of bacteria but negatively with *Chloroflexi*. Soil EC and AP were positively correlated with *Proteobacteria* and *Gemmatimonadetes*. SOC, TN and AN were positively correlated with Shannon index and Chao 1 index of fungal community. TP was positively correlated with *Mortierellomycota. Basidiomycota* was negatively correlated with SOC, TP, and AN. Soil pH was negatively correlated with the Shannon index and Chao 1 index of the bacterial and fungal community (*p* < 0.05, Figure 5).

**Figure 6:**
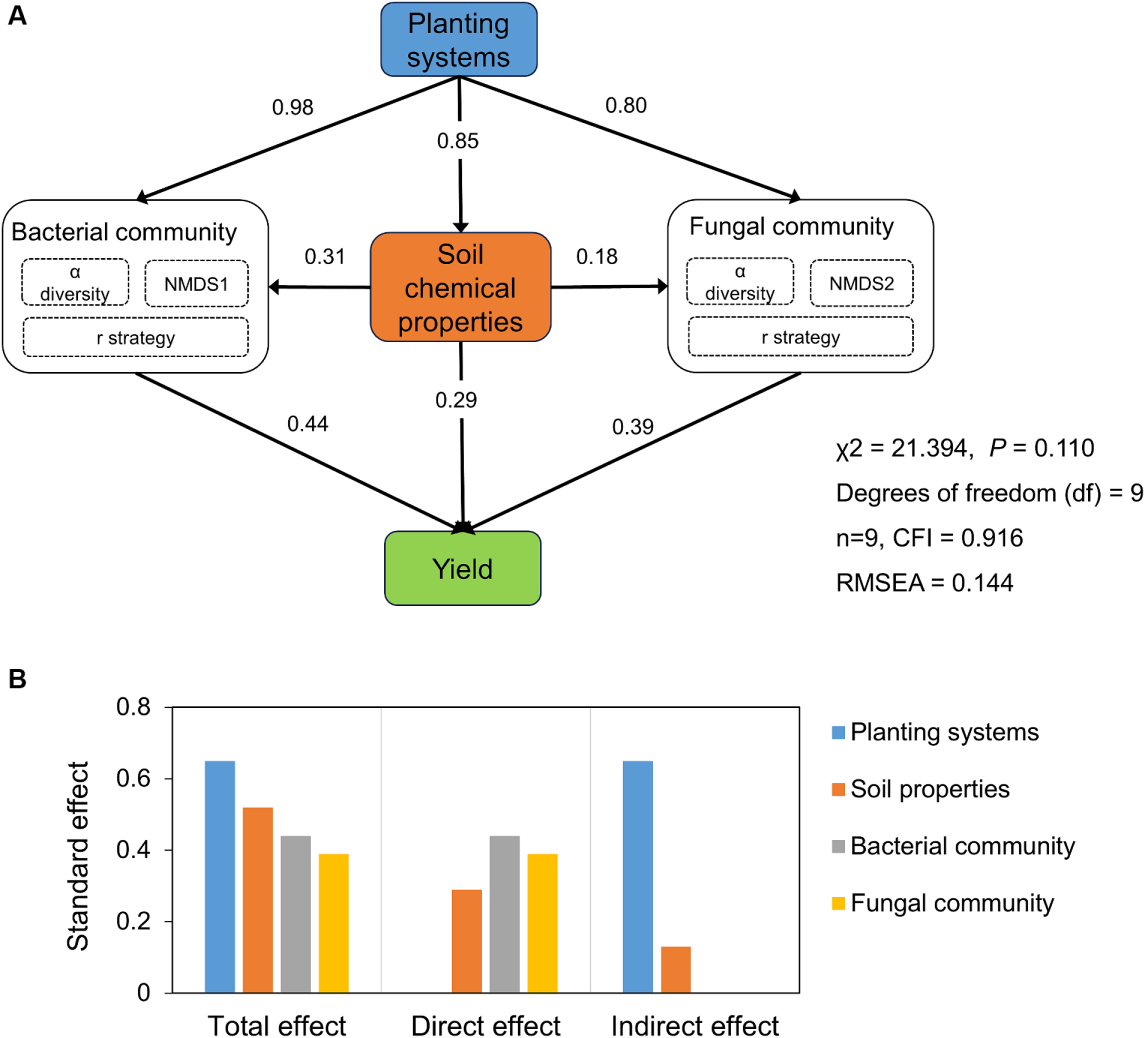
Structural equation modeling (SEM) was conducted to reveal hypothetical pathways between microbial communities, soil chemical properties and yield **(A)**. And standard total, direct and indirect effects of planting systems, microbial communities and soil properties on yield **(B)**.

The effects of planting systems, soil chemical properties, microbial diversity, and community composition on tobacco yield showed that the planting systems had the greatest standard total effect on tobacco yield, followed by soil chemical properties (Figure 6). Soil chemical properties such as SOC, TN, TK, AN, and AK were positively correlated with tobacco yield (*p* < 0.05, Figure 5). Microbial communities, especially bacteria, had the greatest direct effect on tobacco yield (Figure 6). The relationship between tobacco yield and microbes showed that tobacco yield was positively correlated with the Chao 1 index of bacterial and fungal communities, but negatively with *Chloroflexi* and *Basidiomycota* (Figure 5).

## 4 Discussion

### 4.1 Tobacco-rape rotation increased tobacco yield by improving chemical properties

Continuous cultivation of a single crop induced nutrient imbalance, acidification, and autotoxicity (Hati et al., 2007; Pervaiz et al., 2020). But with the increase of above-ground diversity, the diversity of subsurface plant residual inputs enhanced soil ecological services and nutrient cycling under crop rotation system (Karlen et al., 2006; Zuber et al., 2015). In this study, compared with TC, TR increased SOC and soil nutrients including TN, TK, AN, and AK (*p* < 0.05, Table 1). The increased SOC and soil chemical nutrients under the TR can be attributed to the following reasons: (1) rape root residues and exudates entered the soil, expanding the soil C and N pools, regulating the soil C/N, and promoting the formation of SOC (Fang et al., 2021; Li et al., 2021); (2) the large and deep roots of rape can secrete a variety of organic acids and phenolic substances, which have allelopathic effects to dissolve chemically soil minerals and increase their availability as active nutrients (Yang, 2006; Chen and Weil, 2010); (3) rapes as a previous season crop produced a larger surplus of N and P for subsequent crop and reduced the K deficit. A meta-analysis by Zhu et al. (2019) showed that whether or not rape straws were returned, rape rotation produced higher annual nutrient budgets than other crop rotations such as wheat. Compared with TC, TR decreased soil pH (Table 1), which was similar to the results of Jin et al. (2024) and Wang et al. (2008). The result may be attributed to the organic acids secreted by rape roots and the application of nitrogen fertilizer in rape season (Li et al., 2008; Raza et al., 2020). Although TR decreased soil pH, this decrease of pH did not inhibit tobacco growth due to tobacco’s preference for weakly acidic environments and pH in the suitable range for tobacco growth (Ho et al., 2017). In summary, the findings indicated that TR serves as a transformative practice, significantly improving soil chemical properties because of the specificity of rape when compared to TC. It is worth noting that the improvement of soil chemical properties promoted tobacco yield by directly improving the nutrient cycling in the rhizosphere of tobacco and indirectly affecting the microbial diversity and composition (Figure 6). In particular, SOC, TN, TK, AN, and AK significantly increased tobacco yields (Figure 5). Previous studies have shown that planting rapeseed has beneficial effects on the next crop in the rotation cycle, and the rice yield in the rape–rice rotation was 7.5 and 6.4% higher than those in the wheat–rice and fallow–rice rotations, respectively (Huang et al., 2013; Fang et al., 2021). Therefore, due to the specificity of rape, TR was of great significance to increase tobacco yield by improving soil fertility and nutrient cycling.

### 4.2 Tobacco-rape rotation directly and indirectly affected bacterial and fungal communities

Soil microbial communities played a pivotal role in executing the biological processes essential for sustaining soil health and thwarting crop diseases (Garbeva et al., 2004; Raaijmakers et al., 2009). Recent investigations highlighted the critical role of microbial α-diversity in mitigating soil-borne issues. Root exudates were potential drivers of changes in microbial α-diversity. The rotation system appeared to mitigate specific microorganisms influenced by root exudates from the same crop, thereby augmenting microbial diversity (Strickland et al., 2015; Wu et al., 2019). Compared with continuous cropping, rotation reduced specific microorganisms and increased microbial α-diversity by directly avoiding long-term exposure of microorganisms to root exudates from the same crop. Our result also showed that TR increased bacterial and fungal α-diversity compared to TC (*p* < 0.05, Figures 1A–F). Moreover, crop rotation may directly affect microbial community composition by increasing spatio-temporal diversity and crop diversity (Tiemann et al., 2015). Except for reducing imbalances in microbial community structure by changing crop types like other crop rotation systems, rape roots could secrete antimicrobial substances which played a dual role in disrupting the host-pathogen cycle and inhibiting pathogen growth in TR system. For example, the rape root exudates had positive effect on the biocontrol of black shank caused by *phytophthora parasitica* var. *nicotianae* (Fang et al., 2016). Our results support this, with TR promoting beneficial bacterial and fungal taxa (Figure 2). Compared with TC, TR increased the beneficial bacterial taxa including *Sphingomonas, Pyrinomonadaceae*, and *Micrococcaceae* and beneficial fungal taxa including *Nectriaceae*, *Didymellace*, and *Lasiosphaeriaceae* (Behnke et al., 2021). *Sphingomonas* was used as a potential indicator of sustainable management (Li et al., 2022). TR reduced the pathogen that causes root rot, such as *Fusarium* (Supplementary Figure S1; Yao et al., 2020). In addition, the effect of planting systems on microbial co-occurrence network showed that TR increased the nodes and edges for bacteria and fungi, meaning that TR increased the network scale and potential interaction between OTUs (Figure 3). And TR promoted synergies between bacterial taxa and reduced competition (Supplementary Table S3; Figure 3), and the result was consistent with Chen et al. (2018). Therefore, TR may have a direct positive effect on microbial diversity and balancing community structure by changing above-ground crop types and subsurface root exudates.

TR may affect microbial diversity and community structure by improving soil chemistry. In this study, bacterial and fungal α-diversity was positively linked to the soil chemical properties (Kuramae et al., 2012). Specifically, TN, AN were positively correlated with the Shannon index and Chao1 index of bacteria (*p* < 0.05, Figure 5). Similarly, SOC, TN, AN were positively correlated with the Shannon index and Chao1 index of fungi (*p* < 0.05, Figure 5). Sun et al. (2023) indicated the positive effect of N availability on microbial diversity. This suggested that the increase in soil fertility and availability of chemical nutrients under TR promoted the α- diversity of bacteria and fungi. And the overall changes in microbial community structure may be related to soil chemistry driven by planting systems (Bastida et al., 2008). In this study, RDA analysis suggested that pH, SOC, TN, AN, and AK had significant effects on the bacterial and fungal community structure (Figures 4A,B). Notably, AK was the most important factor affecting bacterial community (Figure 4A). Soil AK played an indispensable role in optimizing soil bacterial community structure and advancing bacterial community diversity (Zhao et al., 2022). The bacterial groups, such as *Bacillus mucilaginosus*, *Bacillus edaphicus*, *Bacillus circulans*, and *Acidithiobacillus ferrooxidans* were related to AK (Meena et al., 2014). Also, AK contributed to the synthesis and metabolism of tobacco chemical components (Hu et al., 2021). TN emerged as the predominant factor influencing fungal community composition (Figure 4B), which may be related to the fact that TN affects the environmental stress tolerance of fungi and trade-off with traits enabling organic matter decomposition (Moore et al., 2021).

In addition, soil chemistry influenced microbial community composition through changing microbial life history strategies. For bacterial community composition, compared with TC, TR increased copiotrophs, such as *Proteobacteria*, *Actinobacteria*, and *Gemmatimonadetes*, but decreased oligotrophs including *Chloroflexi* and *Verrucomicrobia*. The effect of crop rotation on microbial life history strategies has also been demonstrated in fungal communities. TR increased the relative abundance of Ascomycota belonging to copiotrophs (Yao et al., 2017). Such changes in microbial life history strategies were related to the improvement of soil chemical nutrient availability. Our results showed that *Proteobacteria* and *Gemmatimonadetes* positively correlated with AP, while *Chloroflexi* negatively correlated with TN and AN (Figure 5). These results showed that copiotrophs grew faster in resource-rich environment, whereas oligotrophs grew slower in resource-poor environment (Fierer et al., 2007; Ho et al., 2017). These findings underscored that long-term regulation of TR on soil chemical properties alleviated microbial nutrient resource constraints and may change soil microbial diversity and community composition, and thus influence soil trade-off and functional stability (Panettieri et al., 2020).

### 4.3 Microbes were the direct factors driving tobacco yield

In dynamic agricultural ecosystems where species turnover occurs across space or time, a diverse soil microbial community as a reservoir of specialized microbial taxa, contributed to soil trade-offs and crop yield (Zhang et al., 2023). The efficacy of TR in bolstering tobacco yield was evident compared to TC (*p* < 0.05, Table 1). Assessing the factors influencing yield revealed a hierarchy with planting systems exerting the greatest total effect (Xuan et al., 2012), followed by soil chemical properties, bacterial community, and fungal community. Within this intricate web of influences, the bacterial community emerged as a key player, exerting the greatest direct effect on tobacco yield (Figure 6). This underscored the pivotal role of microbial communities in shaping the soil environment and influencing crop productivity (Ghani et al., 2022; Jiang et al., 2022). Our results showed that the α-diversity of bacteria and fungi was positively correlated with tobacco yield (Figure 5). On the one hand, soils with high microbial diversity may accelerate the turnover rate of nutrients, which implied that microbial diversity facilitated more nutrients for plant growth and that there was less competition from microbes (Fan et al., 2021). On the other hand, high microbial diversity may confer protection against soil-borne diseases, and ultimately increased plant productivity (Xuan et al., 2012). Moreover, the changes in microbial composition associated with increased crop yields are characterized by responses from potentially beneficial bacteria. For instance, the increased *Actinomycetes* under tobacco-rape rotation were symbionts of plants and saprophytes (Bhattacharyya and Jha, 2012), and played a role as biocontrol agents against a range of pathogenic fungi and promote plant growth (Franco-Correa et al., 2010; Bhattacharyya and Jha, 2012). In summary, this investigation unraveled the intricate interplay among yield, microbial communities, and soil chemical properties driven by planting systems. These findings highlighted the potential of microbial communities to drive crop yield increases in tobacco-rape rotation.

## 5 Conclusion

The study demonstrated the potential of tobacco-rape rotation to increase crop yield by directly and indirectly improving microbial diversity and optimizing microbial community structure. Compared with tobacco continuous cropping, tobacco-rape rotation increased the Shannon index, Chao1 index, and ACE index of bacteria and fungi, indicating the increase of microbial α-diversity. On the one hand, the tobacco-rapeseed rotation may directly affect the bacterial and fungal community structure due to the specificity of crop type and root exudates. The results showed that tobacco-rape rotation increased beneficial bacterial taxa including *Sphingomonadaceae*, *Pyrinomonadaceae*, and *Micrococcaceae* and the beneficial fungal taxa including *Nectriaceae*, *Didymellace*, and *Lasiosphaeriaceae*. At the same time, tobacco-rape rotation increased the nodes and edges of bacteria and fungi, implying increased co-occurrence network size and complexity, and promoted potential synergies or cooperative relationships between bacterial OTUs. On the other hand, tobacco-rape rotation indirectly changed the composition of microbial community by improving soil chemical properties and regulating microbial life history strategies. The results showed that tobacco-rape rotation increased the relative abundance of copiotrophs including *Proteobacteria*, *Actinobacteria*, *Gemmatimonadetes*, and *Ascomycota*, while simultaneously reduced oligotrophs including *Chloroflexi* and *Verrucomicrobia*. The correlation between soil chemistry and copiotrophs or oligotrophs provided further evidence of the effect of tobacco-rape rotation on microbial community composition by increasing the availability of soil nutrient resources. The relationships among yield, microbial community, and soil chemical properties indicated that planting systems had the greatest total effect on tobacco yield. The microbial community, especially bacteria, that was directly or indirectly regulated by planting systems had the greatest direct effect on tobacco yield. Our findings highlighted the critical role that tobacco-rape rotation played in shaping soil nutrient environments, optimizing microbial community structure, and thus promoting crop productivity.

## Data availability statement

The datasets presented in this study can be found in online repositories. The names of the repository/repositories and accession number(s) can be found here: https://www.ncbi.nlm.nih.gov/, accession numbers: PRJNA1112613 and PRJNA1112749.

## Author contributions

LL: Writing – original draft, Software. QM: Writing – review & editing, Data curation. YG: Writing – review & editing, Validation. CW: Writing – original draft, Investigation. JS: Writing – review & editing, Methodology, Funding acquisition. ZF: Writing – original draft, Investigation. DW: Writing – original draft, Formal analysis. YH: Writing – original draft, Formal analysis. JL: Writing – review & editing, Supervision, Conceptualization. ZC: Writing – review & editing, Visualization, Project administration.
