# Supplementary material for: Bacterial and fungal communities regulated directly and indirectly by tobacco-rape rotation promote tobacco production
Source: Front Microbiol. 2024 Jun 14;15:1418090. doi: 10.3389/fmicb.2024.1418090 (PMC11211276; doi:10.3389/fmicb.2024.1418090)
Supplement: Supplementary file 1 [file Data_Sheet_1.pdf]

Supplementary Material

Table S1 Soil physical properties under tobacco continuous cropping and tobacco-rape rotation.

Planting systems	Soil solid phase (%)	Soil Liquid phase (%)	Soil gas phase (%)	Soil porosity (%)	Soil bulk density (g cm^{-3})
TC	47.9 ± 1 A	32.7 ± 3.37 A	19 ± 4.1 A	52.7 ± 0.93 A	1.25 ± 0.08 A
TR	44.5 ± 3.4 A	35.4 ± 8.9 A	20.1 ± 6.5 A	56.3 ± 3.37 A	1.21 ± 0.1 A

[a] TC, tobacco continuous cropping; TR, tobacco-rape rotation.

Table S2 Main chemical contents related to tobacco quality under tobacco continuous cropping and tobacco-rape rotation.

Planting systems	Total sugar (%)	Reducing sugar (%)	Total nitrogen (%)	Alkaloids (%)	Potassium (%)	Cl (%)	Protein (%)	Sugar/ Alkaloids	Nitrogen/ Alkaloids
TC	324 A	295 A	18.2 A	21.3 A	20.5 A	2.5 A	90.8 A	15.2 A	0.85 A
TR	344 A	309.A	17.7 A	16.4 A	18.7 A	2.1 A	92.8 A	21.3 A	1.09 A

[a] TC, tobacco continuous cropping; TR, tobacco-rape rotation.

Table S3 Effects of tobacco continuous cropping and tobacco-rape rotation on topological properties of bacterial and fungal networks [a].

Microbe	Bacteria		Fungi	
Treatment	TC	TR	TC	TR
Nodes	1217	1354	255	274
Edegs	2638	5971	413	516
Average degree	4.335	8.82	3.239	3.766
Network diameter	24	26	11	16
Average path distance	7.466	9.164	3.707	6.318
Average clustering coefficient	0.339	0.405	0.421	0.386
Modularity	0.722	0.561	0.874	0.782
Density	0.004	0.007	0.013	0.014
Positive links	2400	5813	372	455
Negative links	238	158	41	61
Proportion of positive links (%)	91%	97%	90%	88%

[a] TC, tobacco continuous cropping; TR, tobacco-rape rotation.

Table S4 Redundancy analysis (RDA) demonstrating the relationships between soil properties and bacterial (a) or fungal (b) community structures [a].

Variable	Bacteria			
	R^2	*P*	RDA1	RDA2
pH	0.7351	0.001	-0.7994	-0.6008
EC	0.5015	0.007	-0.5587	0.8294
SOC	0.4129	0.017	0.9994	0.0341
TN	0.7999	0.001	0.9891	0.1474
TP	0.1752	0.248	0.726	0.6877
TK	0.3127	0.064	0.7713	-0.6365
AN	0.7724	0.001	0.9294	0.3691
AP	0.6325	0.001	-0.7034	0.7108
AK	0.8755	0.001	-0.8809	0.4732
Variable	Fungi			
	R^2	*P*	RDA1	RDA2
pH	0.4126	0.017	-0.9489	0.3155
EC	0.1556	0.261	-0.8767	-0.481
SOC	0.4677	0.018	0.9901	-0.1404
TN	0.7913	0.001	0.9974	-0.072
TP	0.2538	0.103	0.3984	-0.9172
TK	0.1668	0.268	0.7015	0.7126
AN	0.7198	0.001	0.9993	-0.0384
AP	0.1227	0.368	-0.9499	-0.3125
AK	0.6291	0.001	-1	-0.0066

[a] EC: electric conductivity; SOC: soil organic carbon, TN: total nitrogen, TP: total phosphorus, TK: total potassium, AN: alkaline hydrolysis nitro-gen, AP: available phosphorus, AK: available potassium.

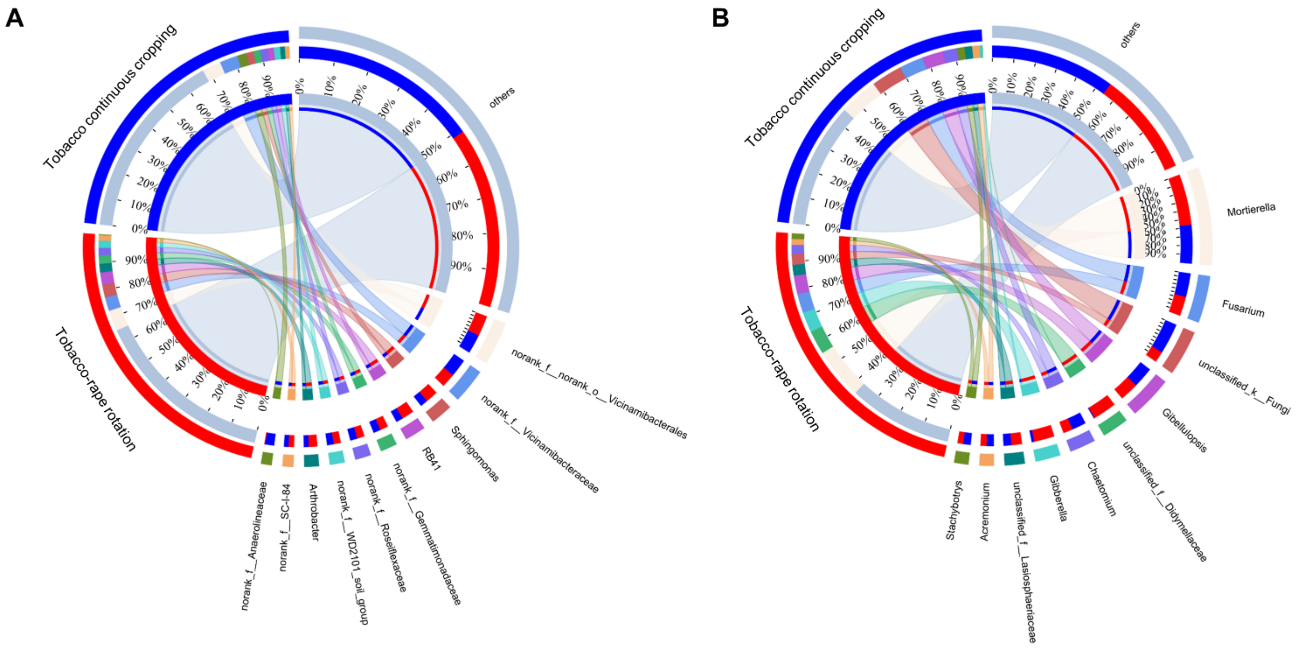

Figure S1. The relative abundance of bacterial (A) and fungal (B) at dominant genus (top 10 in relative abundance) under tobacco continuous cropping (TC) and tobacco-rape rotation (TR).
